# Genotypic detection of rifampicin and isoniazid resistant Mycobacterium tuberculosis strains by DNA sequencing: a randomized trial

**DOI:** 10.1186/1476-0711-8-4

**Published:** 2009-01-30

**Authors:** Amina Abdelaal, Hassan Abd El-Ghaffar, Mohammad Hosam Eldeen Zaghloul, Noha El mashad, Ehab Badran, Amal Fathy

**Affiliations:** 1 Clinical pathology department, Faculty of Medicine, Mansoura University, Mansoura, Egypt; 2 Thoracic medicine department, Faculty of Medicine, Mansoura University, Mansoura, Egypt

## Abstract

**Background:**

Tuberculosis is a growing international health concern. It is the biggest killer among the infectious diseases in the world today. Early detection of drug resistance allows starting of an appropriate treatment. Resistance to drugs is due to particular genomic mutations in specific genes of Mycobacterium tuberculosis(MTB). The aim of this study was to identify the presence of Isoniazid (INH) and Rifampicin(RIF) drug resistance in new and previously treated tuberculosis (TB) cases using DNA sequencing.

**Methods:**

This study was carried out on 153 tuberculous patients with positive Bactec 460 culture for acid fast bacilli.

**Results:**

Of the 153 patients, 105 (68.6%) were new cases and 48 (31.4%) were previously treated cases. Drug susceptibility testing on Bactec revealed 50 resistant cases for one or more of the first line antituberculous. Genotypic analysis was done only for rifampicin resistant specimens (23 cases) and INH resistant specimens (26 cases) to detect mutations responsible for drug resistance by PCR amplification of rpoB gene for rifampicin resistant cases and KatG gene for isoniazid resistant cases. Finally, DNA sequencing was done for detection of mutation within rpoB and KatG genes. Genotypic analysis of RIF resistant cases revealed that 20/23 cases (86.9%) of RIF resistance were having rpoB gene mutation versus 3 cases (13.1%) having no mutation with a high statistical significant difference between them (P < 0.001). Direct sequencing of Kat G gene revealed point mutation in 24/26 (92.3%) and the remaining 2/26 (7.7%) had wild type KatG i.e. no evidence of mutation with a high statistical significant difference between them (P < 0.001).

**Conclusion:**

We can conclude that rifampicin resistance could be used as a useful surrogate marker for estimation of multidrug resistance. In addition, Genotypic method was superior to that of the traditional phenotypic method which is time-consuming taking several weeks or longer.

## Background

Tuberculosis is a growing international health concern. It is the biggest killer among the infectious diseases in the world today, despite the use of a live attenuated vaccine and several antibiotics. After years of decline, TB has re-emerged as a serious public health problem worldwide, especially with increased drug resistance among MTB strains which hinders the success of TB control programs[1]. Isolation, identification, and drug susceptibility testing of MTB and other clinically important mycobacteria can take several weeks because of its slow growth rate. During the past several years, many molecular methods have been developed which can potentially reduce the diagnostic time from weeks to hours. Early detection of drug resistance allows starting of appropriate treatment, which has an impact on better control of the disease[2]. Resistance to drugs is due to particular genomic mutations in specific genes of MTB. To date, nine genes are known to be linked to resistance to first-line anti-TB drugs; katG, inhA, aphC, and kasA for INH resistance, rpoB for RIF resistance, rpsL and rrs for strptomycin (STR) resistance, embB for ethambtol (EMB) resistance, and pncA for pyazinamid (PZA) resistance. Resistance to multiple drugs is the consequence of an accumulation of mutations[3]. RIF interferes with transcription and elongation of RNA by binding to the β-subunit of RNA polymerase. The rpoB gene encodes RNA polymerase enzyme of MTB. So, any mutation in 81 bp hypervariable region of the rpoB gene results in failure of binding and subsequently resistance[4]. The katG gene encodes mycobacterial catalase peroxidase which is the only enzyme in MTB capable of activating the pro-drug INH to active form. Furthermore, katG gene is involved in detoxification of endogenously generated or exogenously supplied hydrogen peroxide[5].

### Aim of the work

This study was designed to identify the presence of INH and RIF drug resistance in new and previously treated tuberculosis (TB) cases using DNA sequencing.

## Methods

### Patients

This study was carried out on 153 tuberculous patients (83 males and 70 females) with positive Bactec 460 culture for acid fast bacilli. They were selected from Mansoura University Hospitals and Mansoura chest hospital. Drug susceptibility testing on Bactec revealed 50 resistant cases for one or more of the first line antituberculous (Isoniazid, rifampicin, ethambutol and streptomycin). All resistant TB cases were subjected to full history taking especially for past history of similar conditions, intake of antituberculous drugs, clinical and radiological examinations. They were 33 males and 17 females with their ages ranging from 19 to 52 years. Informed written consents were taken from all patients and the study was approved by the Ethics Committee of Mansoura University.

### Definitions

Patients were classified into two groups according to their treatment history at the time of diagnosis: new cases, which included patients who had never received anti-TB treatment or who had received treatment for <4 weeks, and previously treated cases, which included patients who had taken anti-TB drugs for at least 4 weeks [6]. Initial resistance was defined as the presence of drug-resistant *M. tuberculosis *strains in new cases. Acquired resistance was defined as the presence of drug-resistant *M. tuberculosis *strains in patients who were reported to have received anti-TB treatment for >4 weeks. Mono resistance was defined as resistance to only one of the four first-line 8 drugs. Multidrug resistance (MDR) was defined as resistance to at least Isoniazid and rifampicine [7,8].

### Collection of the specimens

Three consecutive spontaneously produced early morning sputum samples were collected from patients with pulmonary TB. Samples were collected in sterile containers to avoid contamination with environmental bacteria e.g. Mycobacterium xenopi.

### Laboratory methods

All resistant TB cases were subjected to detection of tubercle bacilli and anti-tuberculous drug susceptibility testing by radiometric method (BACTEC 460 system, Becton Dickinson microbiology Systems, Cockeysville, M.A. 21030, 800-638-8663).

Genotypic analysis was done only for rifampicin resistant specimens (23 cases) and INH resistant specimens (26 cases) to detect mutations responsible for drug resistance by PCR amplification of rpoB gene for rifampicin resistant cases and KatG gene for isoniazid resistant cases. Finally, DNA sequencing was done for detection of mutation within rpoB and KatG genes.

### Molecular characterization of resistant strains

It included three main steps:

1-DNA extraction to release DNA from mycobacterial cells.

2-Amplification of the target part of gene by PCR and detection by agarose gel electrophoresis.

3-Automated DNA sequencing to detect mutation in comparison with the similar region of a wild strain[9].

#### 1-DNA extraction

QIAamp DNA Mini kit was used to purify total DNA from decontaminated sputum samples (Qiagen Inc. 28159 Avenue Stanford, Valencia, CA 91355. fax 800-718-2056, USA).

#### 2-DNA amplification

### PCR amplification of rpoB & KatG genes

A 157-bp fragment of the rpoB gene, from nucleotide 1846 to 2002 (Gen Bank accession no. U12205) was amplified by using the primers TR9(TCGCCGCGATCAAGGAGT)and TR8(TGCACGTCGCGGACCTCCA). The 200-bp KatG fragment targetting codon 315 from nucleotide 904 to 1103 (Gen Bank accession no. X68081) was amplified by using primers KatGIF(AGCTCGTATGGCACCGGAAC) and KatG4RB(AACGGGTCCGGGATGGTG).

Qiagen taq PCR Master (250 Unit): (2× concentrated, contains polymerase, PCR buffer with 3 mM MgCl_2 _and 400 mM of each dNTP).

#### Procedure

Master mix was prepared as shown in Table 1. The required volume for each sample was multiplied by the number of samples and adding a negative control plus one. The mixture was mixed by vortex and dispensed (50 ul) in each tube. PCR tubes were placed in the thermal cycler under the appropriate amplification conditions (Table 2). Each PCR was preceded by a single denaturation step at 94°C for 4 min. and terminated with a single primer extension step at 72°C for 8 min. The last cycle was followed by cooling to 4°C and holding at this temperature until the tubes were taken out off the machine.

**Table 1:** Amplification master mix

**Component**	**Volume/sample**
Sterile nuclease free water	3 ul
Forward primer	6 ul
Reverse Primer	6 ul
Master mix	25 ul
DNA template	10 ul
Final volume	50 ul

**Table 2:** Amplification conditions.

**Gene**	**Denaturation**	**Annealing**	**Extension**	**No. of cycles**
rpoB	94°C, 1 min.	55°C,1 min.	72°C, 1 min.	40 cycles
Ka G	94°C, 1 min.	61°C, 1 min.	72°C, 1 min.	30 cycles

#### 3-DNA Sequencing

**Figure 1:**
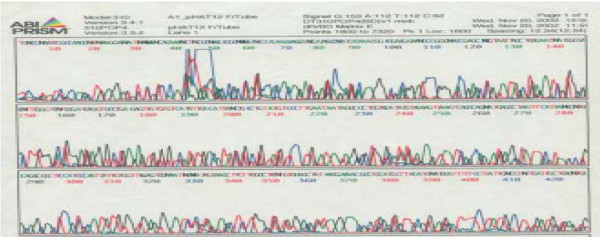
**Sequencing reaction obtained from labeled amplified genes by ABI 310 Sequencer Analyzer**.

This was done by the use of ABI Prism 310 Genetic Analyzer (figure 1), (Applied Biosystems, Foster city, Calif., 944041 USA).

### Statistical analysis

Data entry and analysis were performed using SPSS statistical package for social science version 10 (SPSS, Inc., Chicago, IL, USA). The quantitative data were presented as a mean and standard deviation and the qualitative data were presented as number and percentage. The chi-square (χ2) was used to find the association between row and column variables of qualitative data. The threshold of significance is fixed at 5% level (P value). P value of > 0.05 indicates non-significant results; P value of < 0.05 indicates a significant result while, P value of < 0.001 indicates a high significant result.

## Discussion

Tuberculosis, one of the oldest recorded human diseases, is still one of the biggest killers among the infectious diseases, despite the use of a live attenuated vaccine and several antibiotics. The development of drug resistance in the population has increased the possibility that TB may once again become an incurable disease[10]. Resistance to drugs is due to particular genomic mutations in specific genes of *MTB*. To date, nine genes are known to be linked to resistance to first-line anti-TB drugs; *katG, inhA, aphC*, and *kasA *for INH resistance, *rpoB *for RIF resistance, *rpsL *and *rrs *for STR resistance, *embB *for EMB resistance, and *pncA *for PZA resistance. Resistance to multiple drugs is the consequence of an accumulation of mutations[3].

Because the organism is slow growing, traditional determination of resistance is time-consuming as it may take several weeks or longer. Rapid detection of drug resistance could optimize treatment, improve outcome for patients with drug-resistant tuberculosis and prevent transmission of drug-resistant TB. Genotypic methods have the advantage of a shorter turnaround time, no need for growth of the organism, possibility for direct application in clinical samples, less biohazard risks, and feasibility for automation [8].

Therefore, the aim of this study was to identify the presence of INH and RIF drug resistance in new and previously treated tuberculosis (TB) cases using DNA sequencing. To achieve this aim, 153 tuberculous patients with cultures positive for acid fast bacilli were selected from Mansoura University and Chest Hospitals (Table 3). Out of 153 tuberculous patients, 50 resistant cases to one or more of the first line antituberculous drugs (isoniazid, rifampicine, ethambutol and streptomycin) were selected after drug susceptibility testing by BACTEC 460. All of them were identified by NAP test as Mycobacterium tuberculosis complex. They were 33 males and 17 females with their ages ranging from 19 to 52 years. From 50 resistant cases, genotypic analysis was done only for 23 rifampicine resistant cases (rpoB gene) and 26 isoniazid resistant cases (KatG gene) to detect mutation responsible for drug resistance by automated DNA sequencer (ABI Prism 310 Genetic Analyzer).

**Table 3:** Characteristics of the study population (n = 153)

**Mean age, years (range)**	**38 (19–52)**
Male patients	83 (54.3%)
Female patients	70 (45.7%)
Urban	58 (38%)
Rural	97 (62 %)
Previous TB treatment	48 (31.4%)
New cases	105 (68.6%)
Susceptible TB cases	103(67.3%)
Resistant TB cases	50(32.7%)

The rate of drug resistance (table 4) was 32.7% (50/153 cases), with a higher prevalence of resistance in patients who had received previous anti-tuberculous treatment (70 %) than new patients (30%). This was in accordance with those reported in Saudi Arabia by Al-Hajjaj et al [11] that was 29.7% of 101 MTB isolates. However, a higher prevalence of resistance was found in Russia by Toungoussova et al.[12] that was 56.3% among 119 MTB isolates. Also, in Greece, Tarkada et al.[13] found 51.2% of 207 initial isolates of MTB. While a lower prevalence of resistance was reported by Rivera et al.[14] who studied 188 MTB in Philippines and noted that drug-resistance represented 17.6% of isolates. And in Qatar, by *Al-Marri *[15] who found 61(15%) among 406 MTB isolates. This low prevalence of resistance to four anti-tuberculous drugs may be due to screening programs (chest radiography) and implementation of directly observed therapy, short course(DOTS).

**Table 4:** Previous anti-TB treatment in 50 resistant TB cases

	Resistant TB cases (50)	
	
	No	%
Previous anti-TB treatment(Secondary resistance)	35	70
No previous treatment (Primary resistance)	15	30

Regarding prevalence of primary and secondary resistance, there was a higher prevalence of resistance in patients who received previous antituberculous treatment (70%) than patients with no previous treatment (30%). These results are fully consistent with other reports [4,16,17] stating that resistance was significantly higher in previously treated cases (71%, 63%, and 95%; respectively) than in new cases.

Antituberculous drug susceptibility testing by BACTEC system in the present study (table 5) revealed that INH had the highest resistance (52%), followed by Rifampicine (46%), Ethambutol (44%) and the lowest resistance was encountered with Streptomycin (36%). Also, Dawood [18] found that the drug resistance in TB cases were; 73 %, 63 %, 27 % and zero % to INH, Streptomycin, RIF and ETH; respectively. On the other hand, Annelies et al.[3] reported that resistance was 93%, 56%, 41% and 18% to INH, RIF, STR and EMB; respectively. The significant increase in rifampicin, INH and ethambutol resistance in the last 10 years could be attributed to poor compliance and wide use of these drugs for non-tuberculosis conditions. While, the high sensitivity of streptomycin was attributed to the restriction of its use in the management of tuberculous patients[18]. Furthermore, it could be noticed that the association of RIF resistance with other drugs (table 6, 7) was significantly higher 17/50 (34%) than RIF resistance alone 6/50 (12%). On the other hand, the association of INH resistance with other drugs 16/50 (32%) and INH resistance alone 10/50 cases (20%) was insignificant (P > 0.05). Similar results were found in 1999 by Narar et al.[19] who noted statistical significant difference between resistance to RIF only (5%) and resistant to RIF in association with other drugs (32.5%). So, Albert et al. [20] stated that rifampicine resistance has been identified as a useful surrogate marker for estimation of multidrug resistance and indicated that the second line drugs are urgently required.

**Table 5:** Drug susceptibility testing of resistant TB cases by BACTEC (50 cases)

**Antituberculous drug**	**Sensitive**		**Resistant**	
	
	No	%	No	%
Streptomycin	32/50	64%	18/50	36%
INH	24/50	48%	26/50	52%
Rifampicine	27/50	54%	23/50	46%
Ethambutol	28/50	56%	22/50	44%

**Table 6:** Phenotypic resistance pattern among RIF&INH resistant cases

**Phenotypic resistance pattern**	**No**	**%**	**X2**	**P**
			
RIF resistance alone (Monoresistance)	6/50	12%		
RIF resistance with other drugs (Multidrug resistance)	17/50	34%	0.526	0.022*
				
INH resistance alone (Monoresistance)	10/50	20%	1.38	0.239
				
INH resistance with other drugs(Multidrug resistance)	16/50	32%		

• P value of < 0.05 is significant.

**Table 7:** Different association of drug resistance in studied resistant cases.

**Type of resistance**	**Resistant cases(50)**	
	**No**	**%**
***One drug resistance***	***24***	***46***
*RIF*	6	12
*INH*	10	20
*SM*	4	8
*EMB*	4	8
***Two drug resistance***	***16***	***32***
*RIF+INH*	5	10
*RIF+EMB*	2	4
*INH+SM*	1	2
*INH+EMB*	2	4
*SM+EMB*	6	12
***Three drug resistance***	***7***	***14***
*RIF+INH+SM*	2	4
*RIF+INH+EMB*	3	6
*RIF+ SM+EMB*	2	4
***Four drug resistance***		
*RIF+INH+EMB+SM*	***3***	***6***

Genotypic analysis of RIF resistant cases (table 8) revealed that 20/23 cases (86.9%) showed rpoB gene mutation versus 3 cases (13.1%) having no mutation. A discrepancy between the results of the phenotypic and genotypic drug resistance tests (absence of mutation in 13.1% of phenotypically resistant isolate) could be attributed to the presence of other mutations located either outside the selected target regions (codon position) or outside the selected gene itself (*rpoB *gene). Another explanation was the so called "heteroresistance" that means the presence of mixture of susceptible and resistant subpopulation in culture which could be an obstacle against the sensitivity of molecular drug resistance testing and the successive therapy[21].

**Table 8:** Frequency of mutations among RIF resistant cases (rpo B gene) & INH resistant cases (KatG gene)

	**Mutation**	**No mutation**	**Total**	**X2**	**P**
RIF resistant cases (rpo B)	20 (86.9%)	3 (13.1%)	23(100%)	12.56	<0.001

INH resistant cases (Kat G)	24 (92.3%)	2 (7.7%)	26(100%)	18.16	<0.001

**Figure 2:**
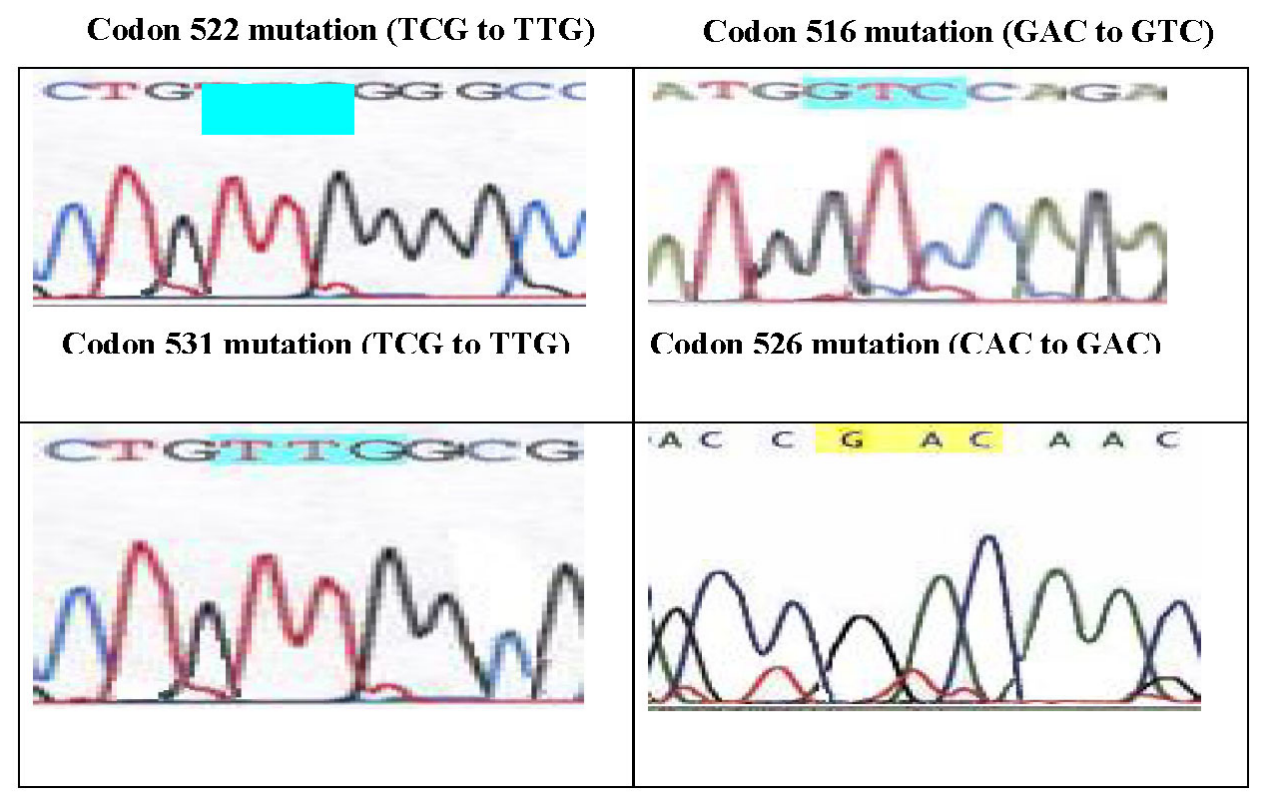
**rpoB gene mutations detected by DNA sequencing in RIF resistant cases**.

The commonest point mutation (table 9 & figure 2) was found at codon 531 in 9/20 (45%) with serine-->leucine substitution (TCG-->TTG). The second type of mutation was detected at codon 526 in 6/20 (30%) with histidine -->aspartate substitution (CAC-->GAC), followed by codon 516 in 4/20 (20%) with aspartate--> valine substitution (GAC-->GTC). The lowest point mutation was found at codon 522 in 1/20 (5%) with serine--> leucine substitution (TCG-->TTG). These results were similar to Kim et al.[22] in Korea, who found high-mutation frequencies of codon 526 (37.8%) and codon 531 (24.4%) in rifampin-resistant strains. Also, these findings were more or less in agreement with those results of the United States[23,24], Japan[25,26], Australia[27], Asia [28]Brazil and France,[29] China,[30] and in Netherlands[31].

**Table 9:** Identification and location of mutation of rpoB gene (20) in RIF resistant cases, and of KatG gene (24) in INH resistant cases

**Mutated gene Codon**	**Nucleotide Substitution**	**Amino acid change**	**Mutation**	
**Mutated rpoB (20)**			No	%
Codon 516	GAC --> GTC	Aspartate -->Valine	4	20
Codon 522	TCG --> TTG	Serine -->Leucine	1	5
Codon 526	CAC --> GAC	Histidine -->Aspartate	6	30
Codon 531	TCG --> TTG	Serine -->Leucine	9	45

**Mutated KatG (24)**				
Codon 315	AGC --> ACC	Serine -->Threonine	24	100

In other similar studies in accordance to our data; Khamis et al.[32] found ten out of the eleven rifampicin resistant specimens (90.9%) showing gene mutation versus only one (9.1%) with no mutation. Point mutations was at codon 516 in 4/10 (40%) with aspargine → valine substitution (CAG → CTG). The second type of mutation was detected at codon 526 in 2/10 (20%) with histidine (H) → tyrosine (Y) substitution (CAC→GAC) and the last mutation was at codone 531 in 4/10 (40%) with serine (S) →leucine (L) substitution (TCG →TTG). Isfahani et al [4] in Iran analyzed 21 RIF resistant isolates and found point mutation in the 81-bp region of the rpoB gene in 18 strains (85.7%) and 3 strains (14.3%) had no mutations. The commonest point mutation was found at codon 531 in 10 cases (47.5%) with serine-->leucine substitution, followed by codon 526 in 4 cases (19.1%) with histidine --> aspargine substitution and codon 516 in one case (4.75%) with aspargine --> valine substitution.

On the other hand, in contrast to the above mentioned studies, Heep et al.[21] had shown a total mutations of 100% at completely unique codon positions apart from codon position mentioned in our study and other studies at codons 176, 441, 451 and 456. This indicates continuous emergence of new codon mutations every now and then.

**Figure 3:**
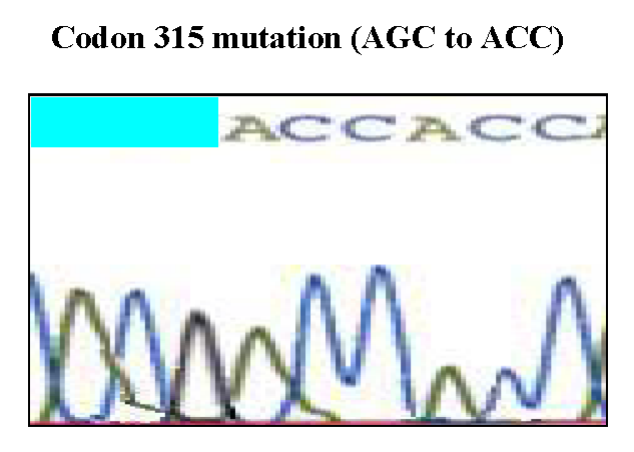
**Identification and location of mutation of KatG gene detected by DNA Sequencing in INH resistant cases**.

Direct sequencing of KatG gene revealed point mutation in 24/26 (92.3%) and the remaining 2/26 (7.7%) had wild type KatG (no evidence of mutation) with high statistical significant difference between them (P < 0.001). Point mutation was found only at codon 315 (figure 3) in 24/24 (100%) with serine --->threonine substitution (AGC-->ACC). Our results had supported the hypothesis of linking *katG *gene mutation to the development of INH resistance in MTB. The *katG *gene encodes mycobacterial catalase peroxidase which is the only enzyme in MTB capable of activating the pro-drug INH to active form. Furthermore, *katG *gene is involved in detoxification of endogenously generated or exogenously supplied hydrogen peroxide[5]. The absence of mutation in 7.7% of resistant isolates could be attributed to possible involvement of other codon positions at the same gene or other genes rather than the studied *katG*.

Our data are in concordance with those reported in the Netherlands by Van Soolingen et al.[31], in Northwestern Russia by Mokrousov et al.[33], in China by Huo and Ge[34], in Lithuania by Bakonyte et al.[17], and in Germany by Hillemann et al [2] who analyzed 103 multidrug-resistant and 40 fully susceptible strains where mutation in *katG *codon 315 was detected in 91 of the 103 MDR strains (88.4%)with (AGC-->ACC) substitution and none of the 40 susceptible strains had mutations.

On the contrary, in Finland, a low prevalence of mutations in codon 315 was detected by Martilla et al.[35] who examined 54 INH-resistant isolates and found 100% katG point mutation with 7.5% only had codon 315 substitution and the remaining mutation positions were at other codons rather than 315. In addition, Ahmad et al.[36,37] found that mutation in codon 315 was detected in 18 (64%) out of 28 isoniazid-resistant isolates from Dubai and in six (35%) out of 17 resistant strains from Beirut. None of the susceptible strains contained this mutation. The genotyping studies showed that the majority of the isolates carrying AGC-->ACC substitution.

## Conclusion

From the previous results, the currently available molecular methods are designed to determine the expected mutations within specific codons of the rpoB and katG genes. Therefore, although the molecular methods may aid in the rapid detection of mutations associated with drug resistance, there are major limitations to the molecular genetic detection of drug resistance; it detects only mutations that are screened for, while phenotypic tests detect resistance independent of the underlying mechanism and not all mutations conferring resistance to anti-TB drugs are known. This fact is especially a problem in the detection of INH resistance because of multiple genes involved. In addition, only few mutations conferring resistance to second-line drugs are known[30].

In summary, rifampicin resistance could be used as a useful surrogate marker for estimation of multidrug resistance and the second line drugs are urgently required. In addition, genotypic method was superior to that of the traditional phenotypic method which is time-consuming taking several weeks or longer. Moreover, the molecular technique, automated DNA sequencing, inspite of its high cost is of value in rapid detection of drug resistance (8 hours) resulting in improving the ability of clinicians to optimize early therapy. It has the advantage of a shorter turnaround time, no need for growth of the organism, possibility for direct application in clinical samples, can detect few numbers of bacilli, less biohazard risks and feasibility for automation. However, Further studies are needed using the automated DNA sequencing technique to identify other codon mutations associated with resistance to other anti-tuberculous drugs to obtain a standardized drug resistance data in Egypt.

## Competing interests

The authors declare that they have no competing interests.

## Authors' contributions

AAA is the one who suggested the idea and participated in the design of the study. HAG, MHZ, NM and EB carried out the molecular biology study and helped to draft the manuscript. AF was responsible for the choice of cases and the clinical data consultation. All authors read and approved the final manuscript.

## References

[B1] Palomino JC (2006). Newer diagnostics for tuberculosis and multi-drug resistant tuberculosis. Curr Opin Pulm Med.

[B2] Hillemann D, Weizenegger M, Kubica T, Richter E, Niemann S (2005). Use of the genotype MTBDR assay for rapid detection of rifampin and isoniazid resistance in Mycobacterium tuberculosis complex isolates. J Clin Microbiol.

[B3] Annelies VR, Warren R, Mshanga I, Jordaan AM, Spuy GD, Richardson M, Simpson J, Gie RP, Enarson DA, Beyers N, van Helden PD, Victor TC (2001). Analysis for a limited number of gene codons can predict drug resistance of Mycobacterium tuberculosis in a high-incidence community. J Clin Microbiol.

[B4] Isfahan BN, Tavakoli A, Salehi M, Tazhibi M (2006). Detection of rifampin resistance patterns in Mycobacterium tuberculosis strains isolated in Iran by polymerase chain reaction-single-strand conformation polymorphism and direct sequencing methods. Mem Inst Oswaldo Cruz.

[B5] Baker LV, Brown TJ, Maxwell O, Gibson AL, Fang Z, Yates MD, Drobniewski FA (2005). Molecular analysis of isoniazid-resistant Mycobacterium tuberculosis isolates from England and Wales reveals the phylogenetic significance of the ahpC-46A polymorphism. Antimicrob Agents Chemother.

[B6] National Tuberculosis Control Program of Egypt Ministry of Health and Population management of tuberculosis. Training for health facility staff.

[B7] World Health Organization Guidelines for the programmatic management of drug-resistant tuberculosis. Geneva: The Organization; Document no WHO/HTM/TB/2006361.

[B8] American Thoracic Society (2000). Diagnostic standards and classification of tuberculosis in adults and children. Am J Respir Crit Care Med.

[B9] Sharma SK, Mohan A (2004). Multidrug-resistant tuberculosis. Indian J Med Re.

[B10] Smith I (2003). Mycobacterium tuberculosis pathogenesis and molecular determinants of virulence. Clinic Microbiol Reviews.

[B11] AL-Hajjaj MS, AL-Kassimi FA, AL-Mobeireek AF, Alzeer AH (2001). Progressive rise of Mycobacterium tuberculosis resistance to rifampicine and streptomycin in Riyadh, Saudi Arabia. Respirology.

[B12] Toungoussova S, Caugant DA, Sandven P, Mariandyshev AO, Bjune G (2002). Drug resistance of Mycobacterium tuberculosis strains isolated from patients with pulmonary tuberculosis in Archangels, Russia. Int J Tuberc Lung Di.

[B13] Tarakada G, Tsiamita M, Spiropoulos K (2004). Drug resistance of Mycobacterium Tuberculosis in Patras, Greece. Monaldi Arch Chest Dis.

[B14] Rivera AB, Tupasi TE, Balagtas ED, Cardano RC, Baello BQ (1999). Drug resistance tuberculosis in the Philippines. Int J Tuber Lung Dis.

[B15] Al-Marri MR (2001). Pattern of mycobacterial resistance to four anti-tuberculosis drugs in pulmonary tuberculosis drugs in pulmonary tuberculosis pateints in the state of Qatar after the implementation of DOTS and a limited expatriate screening programmes. Int J of Tuberc and lung Dis.

[B16] Del-Valle MB, Ponce de-Leon A, Arenas-Huertero C, Vargas-Alarcon G, Kato-Maeda M, Small PM, Couary P, Ruiz-Palacios GM, Sifuentes-Osornio J (2001). rpoB gene mutations in rifampin-resistant Mycobacterium tuberculosis identified by polymerase chain reaction single-stranded conformational polymorphism. Emerg Infect Dis.

[B17] Bakonyte D, Baranauskaite A, Cicenaite J, Sosnovskaja A, Stakenas P (2002). Molecular characterization of isoniazid-resistant Mycobacterium tuberculosis clinical isolates in Lithuania. Antimicrob. Agents Chemother Dis.

[B18] Dawood AM (2000). Value of modern microbiological technique in diagnosis of renal TB. Master degree thesis. Fac Med.

[B19] Nawar NN, Gad WH, Ansary M, Wassef MA (1999). Evaluation of utility of molecular techniques for diagnosing multidrug resistant tuberculosis. MD Thesis in Clinical Pathology, Faculty of Medicine.

[B20] Albert H, Heydenrych A, Brookes R, Mole RJ, Harley B, Subotsky E, Henry R, Azevedo V (2002). Performance of a rapid phage-based test, FAST plaque TB-RIF TM, to diagnose pulmonary tuberculosis from sputum specimens in South Africa. Int J Tuberc Lung Dis.

[B21] Heep M, Brandster B, Rieger U, Lehn N, Richter E, Niemann S (2001). Frequency of rpoB mutations inside and outside the cluster I region in rifampicine-resistant clinical Mycobacterium tuberculosis isolates. J Clin Microbiol.

[B22] Kim BJ, Kim SY, Park BH, Lyu ME, Park IK, Bai GH, Kim SJ, Cha CY, Kook YH (1997). Mutations in the rpoB gene of Mycobacterium tuberculosis that interfere with PCR-single-Strand conformation polymorphism analysis for rifampin susceptibility testing. J Clin Microb.

[B23] Telenti A, Imboden P, Marchesi F, Schmidheini T, Bodmer T (1993). Direct automated detection of rifampin-resistant Mycobacterium tuberculosis by polymerase chain reaction and single-strand conformation polymorphism analysis. Antimicrob Agents Chemother.

[B24] Kapur V, Li LL, Iordanescu S, Hamrick M, Wanger BN, Mooser JM (1994). Characterization by automated DNA sequencing of mutations in the gene rpoB encoding the RNA polymerase B subunit in rifampin resistant Mycobacterium tuberculosis strains from New York City and Texas. J Clin Microbiol.

[B25] Suzuki Y, Katsukawa C, Inoue K, Yin YP, Tasaka H, Ueda N, Makino M (1995). Mutations in rpoB gene of rifampicine resistant clinical isolates of Mycobacterium tuberculosis in Japan. Kansenshogaku Zasshi.

[B26] Ohno H, Koga H, Kohno S, Tashiro T, Hara K (1996). Relationship between rifampin MICs and rpoB mutations of Mycobacterium tuberculosis strains isolated in Japan. Antimicrob Agents Chemother.

[B27] Lilly K, Yuen W, David L, Peter JC (1999). Bacteriological and molecular analysis of rifampin-resistant Mycobacterium tuberculosis strains isolated in Australia. J Clin Microbiol.

[B28] Hirano K, Abe C, Takahashi M (1997). Mutations in the rpoB gene of rifampin-resistant Mycobacterium tuberculosis strains isolated mostly in Asian countries and their rapid detection by line probe assay. J Clin Microbiol.

[B29] Spindola de Miranda S, Kritski A, Filliol I, Mabilat C, Panteix G, Drouet E (2001). Mutations in the rpoB gene of rifampin resistant Mycobacterium tuberculosis strains isolated in Brazil and France. Mem Inst Oswaldo Cruz.

[B30] Yue J, Shi W, Xie J, Li Y, Zeng E, Wang H (2003). Mutations in the rpoB gene of multidrug-resistant Mycobacterium tuberculosis isolates from China. J Clinic Microbiol.

[B31] Van Soolingen D, de Haas PEW, van Doorn HR, Kuijper E, Rinder H, Borgdorff MW (2000). Mutations at amino acid position 315 of the katG gene are associated with high-level resistance to isoniazid, other drug resistance, and successful transmission of Mycobacterium tuberculosis in the Netherlands. J Infect Dis.

[B32] Khamis N, Amin MM, Zagloul MZ, Girgis SA, Shetta M (2004). DNA sequencing and bacteriophage based technique for rapid detection of rifampicine resistant Mycobacterium tuberculosis. Egypt J Med Lab Sci (ESIC).

[B33] Mokrousov I, Narvskaya O, Otten T, Limeschenko E, Steklova L, Vyshnevskiy B (2002). High prevalence of KatG Ser315Thr substitution among isoniazid-resistant Mycobacterium tuberculosis clinical isolates from north-western Russia, 1996 to 2001. Antimicrob Agents Chemother.

[B34] Huo YN, Ge CR (2002). Rapid screening of katG gene mutation in isoniazid-resistant Mycobacterium tuberculosis. Zhejiang Da Xue Xue Bao Yi Xue Ban.

[B35] Martilla JH, Soini E, Erola E, Otten AV, Vasilyef, Viljanen MK (1998). A Ser315Thr substitution in katG is predominant in genetically heterogenous multidrug-resistant Mycobacterium tuberculosis isolates originating from the St. Petersburg area in Russia. Antimicrob Agents Chemother.

[B36] Ahmad S, Fares E, Araj GF, Chugh TD, Mustafa AS (2002). Prevalence of S315T mutation within the katG gene in isoniazid-resistant clinical Mycobacterium tuberculosis isolates from Dubai and Beirut. Inter J Tuber and lung dis.

[B37] Ahmad S, Jaber AA, Mokaddas E (2007). Frequency of embB codon 306 mutations in ethambutol-susceptible and resistant clinical Mycobacterium tuberculosis isolates in Kuwait. Tuberculosis.

