# Genotypic detection of rifampicin and isoniazid resistant Mycobacterium tuberculosis strains by DNA sequencing: a randomized trial

**DOI:** 10.1186/1476-0711-8-5

**Published:** 2009-02-16

**Authors:** Amina Abdelaal, Hassan Abd El-Ghaffar, Mohammad Hosam Eldeen Zaghloul, Noha El mashad, Ehab Badran, Amal Fathy

**Affiliations:** 1 Clinical pathology department, Faculty of medicine, Mansoura university, Mansoura, Egypt; 2 Thoracic medicine department, Faculty of medicine, Mansoura university, Mansoura, Egypt

## Abstract

Correction to Genotypic detection of rifampicin and isoniazid resistant Mycobacterium tuberculosis strains by DNA sequencing: a randomized trial Amina Abdelaal, Hassan Abd El-Ghaffar, Mohammad Hosam Eldeen Zaghloul, Noha El mashad, Ehab Badran, Amal Fathy *Annals of Clinical Microbiology and Antimicrobials *2009, 8:4 (30 January 2009)

## Correction

We lately discovered an accidental mistake, our manuscript [1] needs some important corrections; we offer our sincerest apologies and correct the text as under.

B.15.8 in sentence 2, asparagine is converted to aspartate.

B.15.11 in sentence 2, tyrosine is converted to threonine.

Table 9, asparagine is converted to aspartate, tyrosine is converted to threonine.

## References

[B1] (2009). Genotypic detection of rifampicin and isoniazid resistant Mycobacterium tuberculosis strains by DNA sequencing: a randomized trial Amina Abdelaal, Hassan Abd El-Ghaffar, Mohammad Hosam Eldeen Zaghloul, Noha El mashad, Ehab Badran, Amal Fathy. Annals of Clinical Microbiology and Antimicrobials.

